# Assessing the Impact of Video-based Training on Laceration Repair: A Comparison to the Traditional Workshop Method

**DOI:** 10.5811/westjem.2015.9.27369

**Published:** 2015-10-22

**Authors:** Nicholas Chien, Terren Trott, Christopher Doty, Brian Adkins

**Affiliations:** University of Kentucky, Department of Emergency Medicine, Lexington, Kentucky

## INTRODUCTION

Medical school curricula in the United States have been more recently focusing on the early integration of clinical sciences and clinical experiences into medical students’ pre-clinical years. For many medical students, the common mode of instruction for developing the procedural skill of laceration repair is largely from live workshop training requiring a significant amount of physical resources and physician time to train the students. This study compares the effectiveness of video-based learning (VBL) to traditional live workshop learning (LWL) on student laceration repair performance.

### Review of literature

We performed a review of the literature with searches in PubMed such as video, suturing, medical education, learning, medical students and found several relevant articles published in the last 10 years. Several studies have investigated integrating video modules into medical curriculum and overall, findings have been controversial. One study aimed to identify willingness to learn from video modules in virtual patient encounters. A total of 120 students took a post-encounter survey with majority preferring text-based learning over video. However, the video modules were perceived to be more thorough and with higher detail. In a second study, third-year medical students used VBLs for their pediatrics rotation and video modules were statistically associated with higher recognition of principal symptoms, appropriate diagnosis and consistency between observed symptoms and diagnosis. No studies have been found that used video modules for suturing technique.

## METHODS

We invited first-year medical students at the University of Kentucky College of Medicine to participate in the laceration repair study. Inclusion criteria included students with no prior suturing experience and who were available to attend training (August 26, 2014) and two assessments (September 2 and November 11, 2014). Students were asked to adhere to a set of study rules where they not allowed to discuss the laceration repair study with classmates, attempt to contact other members of the study, and discuss or identify learning resources with classmates. We enrolled the first 40 students to confirm their eligibility and reply via email.

**Figure 1:**
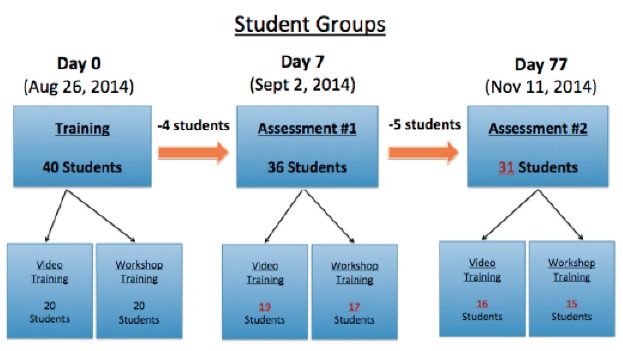
Student groups with randomization to video training and workshop training as on days 0, 7, 77.

Students were randomized into two groups: VBL and LWL (Figure 1). Randomization was performed by assigning students a number, between 1 and 40, based on their order of enrollment. Students were then separated into two groups defined by odd and even assigned numbers. Students with even numbers were assigned to the VBL group, while students with odd numbers were assigned to the LWL group. On the day of training, study participant were provided one banana, one scissor, four Ethicon 4-0 silk sutures, one Addison forcep, and one needle driver as tools to practice suturing techniques. Participants were asked to keep their practice materials for the remainder of the study.

**Figure 2:**
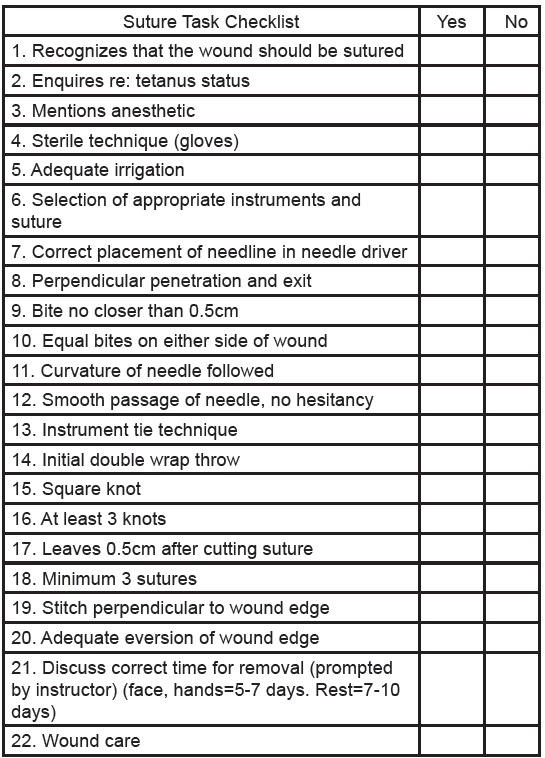
Suture task checklist. Suture Task Checklist derived from: Khan et al. Assessing Surgical Skill Using Bench Station Models. Conner HM and McGraw RC. Clinical skills training: developing objective assessment instruments.

We developed a suture task checklist (Figure 2) by combining various assessment criteria used in the evaluation of suturing as published in Assessing Surgical Skill Using Bench Station Models (Khan et al.) and Clinical skills training: developing objective assessment instruments (Conner, H.M. and McGraw, R.C.). Workshop content was solely based off assessment criteria from the suture task checklist.

The live workshop was presented in Microsoft PowerPoint format by a second-year EM resident and was recorded live using Echo360 lecture capture software. An adjustable camera toggled by the presenter was used to capture imaging of the instructors hands while performing suturing technique. The video recording of the workshop was posted for the 20 students in the VBL group, leaving both groups with the same instructional content.

The live workshop consisted of a 20-minute lecture followed by one hour and 40 minutes of practice and instructional feedback. There were a total of three resident physicians, including the instructor, who provided instructional feedback and tips to students during their allotted practice time. Students were not permitted to ask questions during the lecture as the lecture was being recorded for the video-training arm of the study. Students were free to ask questions during their 1hr 40min practice session and allowed to leave at any time during their practice session.

Two faculty physicians from the University of Kentucky Department of Emergency Medicine, Dr. Christopher Doty and Dr. Brian Adkins, generously volunteered their time to provide mentorship, project oversight, and assessment of student suturing performance using the 22-point suture task checklist. Both physicians were blinded to participant group assignment during the two student assessments and were present for the entirety of each assessment. The average of the two independent numerical values derived from each physician’s 22-point suture task checklist was taken and used as the student’s final score. We used a Welch two Sample t-test to compare performance between groups.

## RESULTS

**Figure 3:**
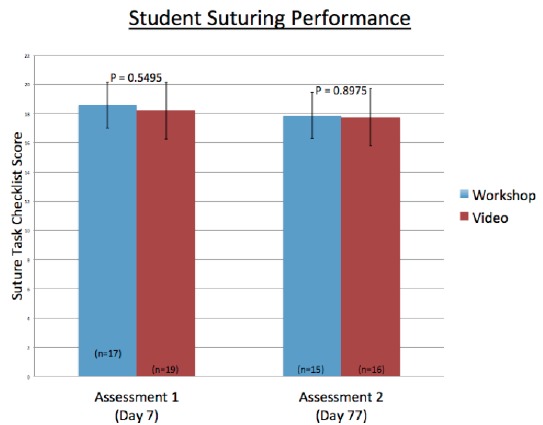
Demonstrates success of suturing performance based on 22-point check list as evaluated on days 7 and 77.

For the first assessment, 36 students were evaluated. The LWL group (n=17) scored a mean of 18.59 (SD 1.8, 95% CI [17.6–19.3]); while the VBL group (n=19) scored a mean of 18.21 (SD 1.8, 95% CI [17.3–19.0]) (p-value 0.549) (Figure 3). For the delayed assessment, 31 students were evaluated. The LWL (n=15) scored a mean of 17.87 (SD 2.5, 95% CI [16.6–19.1]); while the VBL group (n=16) score a mean of 17.75 (SD 2.5, 95% CI [16.6–19.0]) (p-value 0.8979).

Evaluator concordance using 22-point suture task checklist was as follows: Evaluators’ assessment scores were identical 29.9% of the time; evaluators’ assessment scores differed by one point 44.8% of the time. Therefore, evaluators scored students within one point of each other 74.7% of the time.

## DISCUSSION

Medical students often use shadowing experiences, simulation labs, and live workshops to develop procedural skills such as laceration repair that will better prepare themselves for their clinical rotations. Many of these experiences require a tremendous amount of training resources (physician time, space, practice materials, and live tissues) and planning to synchronize the availability of students and physicians. In our study, students who participated in VBL had no significant difference in suturing scores at one and three months compared to LWL. These results suggest that VBL may be as effective as live workshop training. The implementation of accessible VBL into medical students’ pre-clinical education may be an effective way to teach students procedural skills while saving time, space, and resources used for scheduled instruction in an environment of ever-increasing educational demands.

While VBL serves as a promising educational tool, some limitations to this mode of learning include limited interaction with residents and physicians and lack of instructor feedback. Limitations to this study include not including baseline/pre-intervention evaluation of subjects suturing skillsets, small sample size, and the quality of the overhead camera used to capture suturing techniques and ties may not have been optimal for high resolution viewing at home. Future studies may look to evaluate VBL performance beyond a controlled practice environment and into real-life clinical situations. In addition, student preference between VBL and LWL should be assessed.

### Grant Citation

The project described was supported by the National Center for Advancing Translational Sciences, through Grant UL1TR000117. The content is solely the responsibility of the authors and does not necessarily represent the University.
